# Interactional mechanisms of *Paenibacillus polymyxa* SC2 and pepper (*Capsicum annuum* L.) suggested by transcriptomics

**DOI:** 10.1186/s12866-021-02132-2

**Published:** 2021-03-04

**Authors:** Hu Liu, Yufei Li, Ke Ge, Binghai Du, Kai Liu, Chengqiang Wang, Yanqin Ding

**Affiliations:** College of Life Sciences, Shandong Engineering Research Center of Plant-Microbia Restoration for Saline-Alkali Land, Shandong Key Laboratory of Agricultural Microbiology, National Engineering Laboratory for Efficient Utilization of Soil and Fertilizer Resources, Shandong Agricultural University, Tai’an, 271018 China

**Keywords:** *Paenibacillus polymyxa*, *Capsicum annuum*, PGPR, Interaction mechanisms, RNA-seq

## Abstract

**Background:**

*Paenibacillus polymyxa* SC2, a bacterium isolated from the rhizosphere soil of pepper (*Capsicum annuum* L.), promotes growth and biocontrol of pepper. However, the mechanisms of interaction between *P. polymyxa* SC2 and pepper have not yet been elucidated. This study aimed to investigate the interactional relationship of *P. polymyxa* SC2 and pepper using transcriptomics.

**Results:**

*P. polymyxa* SC2 promotes growth of pepper stems and leaves in pot experiments in the greenhouse. Under interaction conditions, peppers stimulate the expression of genes related to quorum sensing, chemotaxis, and biofilm formation in *P. polymyxa* SC2. Peppers induced the expression of polymyxin and fusaricidin biosynthesis genes in *P. polymyxa* SC2, and these genes were up-regulated 2.93- to 6.13-fold and 2.77- to 7.88-fold, respectively. Under the stimulation of medium which has been used to culture pepper, the bacteriostatic diameter of *P. polymyxa* SC2 against *Xanthomonas citri* increased significantly. Concurrently, under the stimulation of *P. polymyxa* SC2, expression of transcription factor genes *WRKY2* and *WRKY40* in pepper was up-regulated 1.17-fold and 3.5-fold, respectively.

**Conclusions:**

Through the interaction with pepper, the ability of *P. polymyxa* SC2 to inhibit pathogens was enhanced. *P. polymyxa* SC2 also induces systemic resistance in pepper by stimulating expression of corresponding transcription regulators. Furthermore, pepper has effects on chemotaxis and biofilm formation of *P. polymyxa* SC2. This study provides a basis for studying interactional mechanisms of *P. polymyxa* SC2 and pepper.

**Supplementary Information:**

The online version contains supplementary material available at 10.1186/s12866-021-02132-2.

## Background

Plant growth-promoting rhizobacteria (PGPR) inhabit the rhizosphere soil and can effectively promote plant growth through a variety of direct and indirect mechanisms [1]. PGPR directly promote plant growth by solubilizing insoluble phosphate [2, 3], fixing nitrogen [4], producing plant hormone [5], secreting 1-aminocyclopropane-1-carboxylate (ACC) deaminase [6], producing siderophores [7], and so on. Furthermore, PGPR demonstrate antagonistic activities against pathogenic microorganisms by secreting secondary metabolites such as polymyxin [8], surfactin [9], and fengycin [9]. PGPR also indirectly promote plant growth by inducing plant systemic resistance to resist invasion by external pathogens [10, 11].

*Paenibacillus polymyxa*, an important member of the PGPR, is widely distributed in the rhizosphere soil of wheat, maize [12], pepper [13], sorghum [14], pine forest [15], etc., and directly or indirectly improves the growth of numerous plants. *P. polymyxa* WR-2 suppressed growth of *Fusarium oxysporum* f. sp. *Niveum* by producing volatile organic compounds [16]. *P. polymyxa* CR1 enhanced growth of maize, potato, cucumber, *Arabidopsis*, and tomato plants through direct mechanisms such as phosphate solubilization and production of indole-3-acetic acid (IAA) [17]. *P. polymyxa* BFKC01 promoted growth of *Arabidopsis* by secreting IAA and promoting iron acquisition [18]. *P. polymyxa* P2b-2R, an endophytic diazotroph of pine, might facilitate regeneration and growth of western red cedar at nitrogen-poor sites [19]. *P. polymyxa* B2 promoted growth of winter wheat by increasing the available phosphorus in the soil [20]. *P. polymyxa* CF05 promoted growth of tomato seedlings in the greenhouse [21], and *P. polymyxa* SC2 was reported as a plant growth-promoting rhizobacterium isolated from the rhizosphere soil of pepper in Guizhou, China [13]. *P. polymyxa* SC2 has a wide antimicrobial spectrum and antagonistic effects on various plant pathogens [22], including *Fusarium vasinfectum* Atk., *F. oxysporum* f. sp. *cucumerinum*, *Pseudoperonospora cubensis*, *Botrytis cinerea* Pers, and *Botrytis cinerea*. *P. polymyxa* SC2 could promote pepper growth, but the molecular mechanisms underlying the interaction between *P. polymyxa* SC2 and pepper remain unclear.

The development of omics technologies has led to interest in the interaction between PGPR and plants. A metabolomics study showed that *Pseudomonas fluorescens* induced root formation in *Sedum alfredii* by increasing the concentration of IAA and reducing the contents of abscisic acid, brassinolide, trans zeatin, ethylene, and jasmonic acid [23]. Transcriptome analysis of *Arabidopsis thaliana* revealed that aluminum-activated malate transporter (ALMT1) plays an important role in *Bacillus subtilis* FB17 colonization [24]. In response to rice seedlings, 43 genes related to metabolism or transport of carbohydrates or amino acids were significantly expressed in *B. subtilis* OKB105 [25]. Singh et al. reported that *Enterobacter cloacae* SBP-8 increased the tolerance of wheat to salinity stress through regulation of transcription factors, proteins of the Ninja family, and other defense-related enzymes and proteins [26]. These studies demonstrate that by using omics technologies some progress has been made in elucidating the interactions between *Bacillus*, *Enterobacter*, *Pseudomonas,* and plants. However, studies on the interaction between *P. polymyxa* and plants are limited. Kwon et al. reported that *P. polymyxa* E681 increased the concentrations of tryptophan, indole-3-acetonitrile (IAN), IAA, and camalexin in the treated plants, and also activated defense-related proteins against fungal pathogens in plants [27]. Our group previously found that *P. polymyxa* YC0136 promoted the growth of tobacco (*Nicotiana tabacum* L.) by inducing hormone-related genes and systemic resistance genes in tobacco [28]. The present study aimed to understand the molecular mechanisms involved in the interaction between *P. polymyxa* SC2 and pepper by conducting transcriptomic sequencing of co-cultured *P. polymyxa* SC2-pepper samples.

## Results

### Growth promotion characteristics of *P. polymyxa* SC2 on peppers

**Fig. 1:**
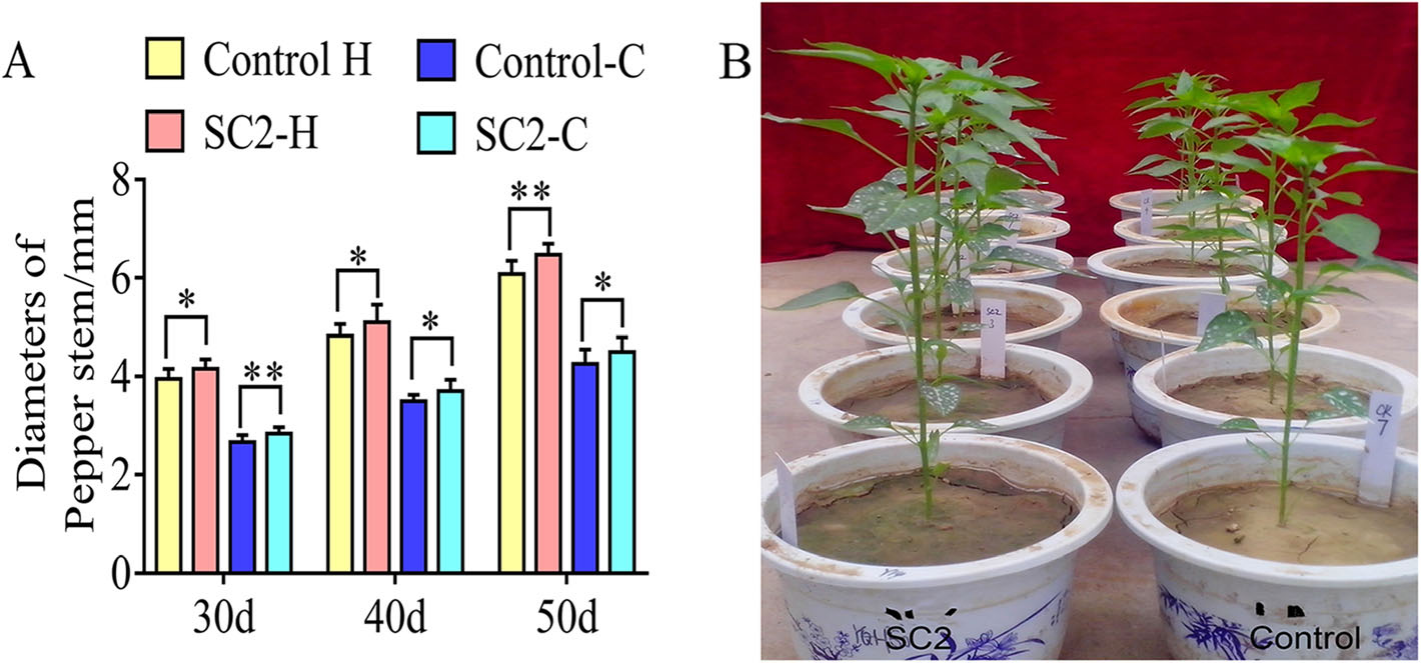
Interaction effects of *P. polymyxa* SC2 on pepper seedlings. In pot experiments, a pepper seedling was irrigated with 5 mL *P. polymyxa* SC2 cells (1 × 10^8^ CFU/mL) and diluted with water to 200 mL. Control plant was irrigated with 5 mL sterilized LB medium diluted with water to 200 mL. At 30, 40, and 50 dpi, stem diameters (diameter at the ground base) were investigated. Panel (**a**) shows pepper stem diameters in healthy (H) soil and continuous cropping (C) soil. Values indicate means ± SD (*n* = 9; * *P* < 0.05, ** *P* < 0.01, Student’s *t*-test). Panel (**b**) is a representative image of the status of pepper in healthy soil at 40 dpi.

To identify growth promotion characteristics of *P. polymyxa* SC2 on pepper, pot experiments in healthy soil and continuous cropping soil were performed in the greenhouse. In healthy soil, there were distinct differences in pepper growth between the *P. polymyxa* SC2-treated group and the control group. At 30- and 40-days post-inoculation (dpi), stem diameters of peppers inoculated with *P. polymyxa* SC2 were significantly thicker than those of the control group, with increases of 5.26 and 5.7%, respectively (*P* < 0.05; Fig. 1a). At 50 dpi, there was an extremely significant difference (*P* < 0.01) in the stem diameter of peppers, with a 6.52% increase in the *P. polymyxa* SC2-treated group compared with the control group. The growth status of pepper treated with *P. polymyxa* SC2 in healthy soil at 40 dpi was shown in Fig. 1b. At 40 dpi, the indices of pepper leaves were evaluated (Table 1). The width and length of leaves in the *P. polymyxa* SC2-treated group were 6.1 and 4.51% larger, respectively, than those of the control group. There was a significant difference (*P* < 0.05) in chlorophyll content between the two treatment groups. Chlorophyll content in the *P. polymyxa* SC2-treated group increased by 14% compared with the control group. These results indicated that *P. polymyxa* SC2 promoted the growth of pepper in healthy soil.

**Table 1:** Indices of pepper leaves in healthy soil at 40 dpi

Treatment	Leaf length /cm	Leaf width /cm	Chlorophyll content / mg·g^− 1^
Control	10 ± 0.67 a	4.45 ± 0.31 a	2.0277 ± 0.13178 a
Strain SC2	10.61 ± 0.85 b	4.66 ± 0.33 a	2.3115 ± 0.09624 b

Note: Values indicate mean ± SD (*n* = 5). The different lower-case letters (a, b) indicate differences at the level of *P* < 0.05 in Student’s *t*-test

In the continuous cropping soil, *P. polymyxa* SC2 also promoted the growth of pepper, as evidenced by increases in the diameters of pepper stems at different harvest intervals (Fig. 1a). At 30 dpi, stem diameters in the *P. polymyxa* SC2-treated group were 6.4% thicker than the control group (*P* < 0.01), and at 40 and 50 dpi, the differences in pepper stem diameters between the two treatment groups were significant at the level of *P* < 0.05.

### Transcriptome analysis of co-cultured *P. polymyxa* SC2 and pepper

To study the molecular mechanisms involved in the interaction of *P. polymyxa* SC2 and pepper, RNA-seq of *P. polymyxa* SC2 and pepper co-cultured under a sterile environment was performed. After sequencing, about 156,499,912 and 158,836,654 raw reads were obtained in the pepper control group (marked as P) and pepper treated group (marked as PH) respectively. And about 72,827,434 and 92,447,218 raw reads were generated in the strain SC2 control group (labeled as S) and strain SC2 treated group (labeled as SH). For *P. polymyxa* SC2 and pepper, 151,205,362 and 308,431,888 high-quality sequences were generated, respectively. Mapping of the transcriptome sequences with the whole-genome sequence of *P. polymyxa* SC2 indicated that the mapped percentage of each sample was more than 90% (the mapping proportion statistics was shown in Additional File 1: **Table S1**). In *P. polymyxa* SC2, 5014 genes mapped with the reference genome. The percentage of mapped genes in each pepper sample was higher than 85% (the mapping proportion statistics were shown in Additional File 1: **Table S2**). We also carried PCA analysis, and the results were shown in Additional File 2: **Fig. S1**. All results met the requirements for subsequent analyses.

**Fig. 2:**
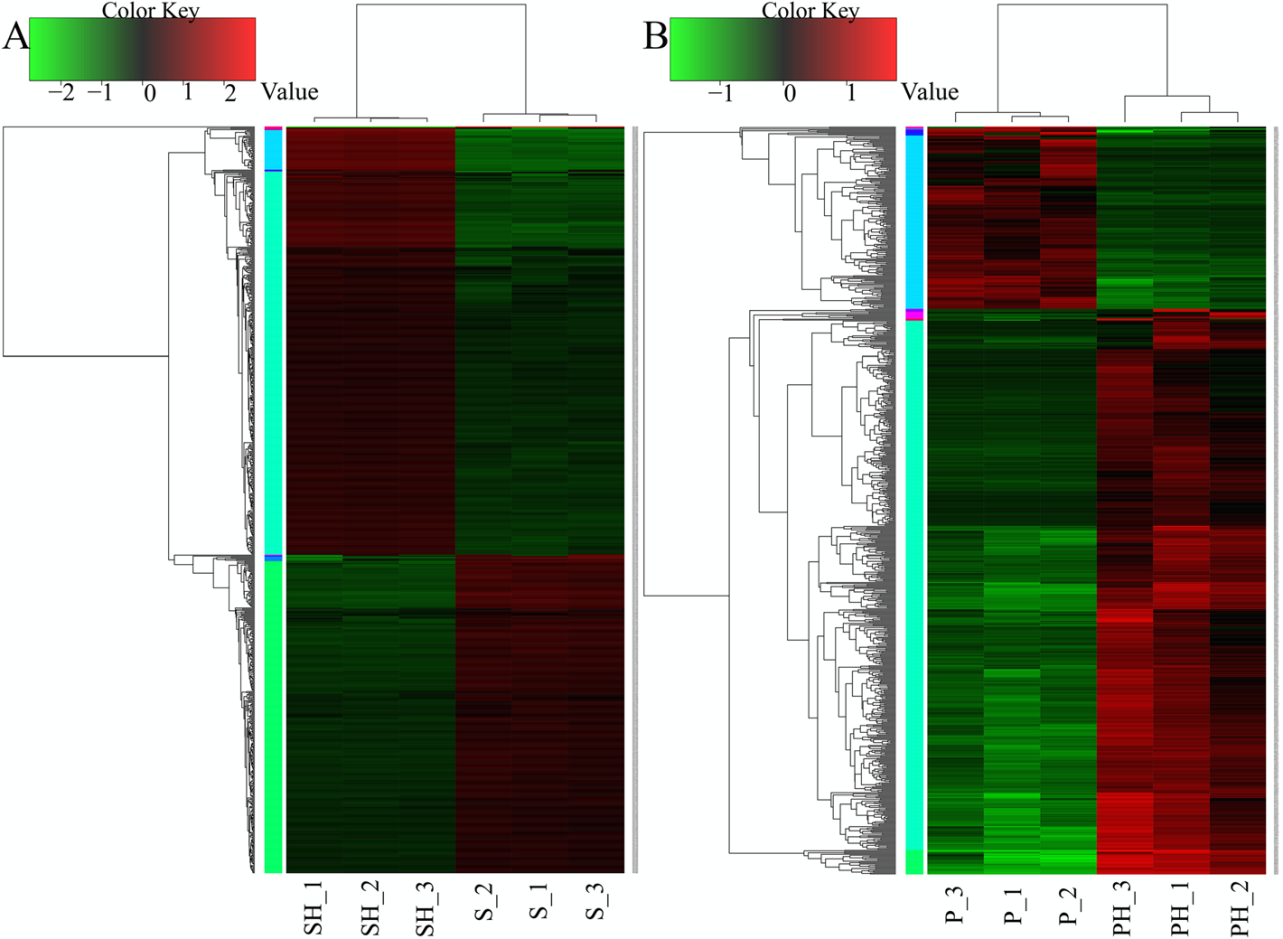
Heatmap of DEGs in *P. polymyxa* SC2 (**a**) and pepper (**b**). Cluster analysis of expression patterns of genes/transcripts with significant differences was performed using a distance calculation algorithm. Spearman’s correlation coefficient was used to analyze the correlation among samples, while Pearson’s correlation coefficient was used for gene correlation analysis, and the cluster method was hcluster (complete algorithm). Each column in the figure represents a sample, and each row represents a gene. The color represents the gene expression in the group of samples (log10 FPKM); red indicates the gene is highly expressed in this sample, and green represents low expression. The number label under the colored bar at the top left of each panel presents the specific values for changes in gene expression. For each panel, the dendrogram of gene clustering is on the left, and the gene name is on the right. The closer the two gene branches are in the dendrogram, the closer their expression levels. The upper part of each heatmap depicts the dendrogram of sample clustering, while sample names are at the bottom of each heatmap. The closer the branches of two samples are in this dendrogram, the closer the expression patterns of all genes in these two samples. The original figures were shown in additional files (Additional File 5: **Fig. S2,** Additional File 6: **Fig. S3)**

Differentially expressed genes (DEGs) of *P. polymyxa* SC2 were detected based on the criterion of *p*-value< 0.05 and |log_2_FC| > 2, while DEGs of pepper were detected according to *p*-value< 0.05 and |log_2_FC| > 4. Genes with significantly up-regulated and down-regulated expression are shown in Fig. 2. In *P. polymyxa* SC2, there were 812 DEGs, of which 465 were up-regulated and 347 were down-regulated (Fig. 2a). Annotation information for these DEGs is displayed in Additional File 3: **Table S3.** The most significantly up-regulated genes of *P. polymyxa* SC2 were involved in polymyxin biosynthesis, fusaricidin biosynthesis, phosphatase/MFS transporter, acetolactate synthase, and 3-hydroxydecanoyl dehydratase, etc. The down-regulated significant genes were related to D-ribose transport subunit RbsB, Ribose ABC transporter, membrane protein, oxidoreductase and stress protein, etc. In pepper (*Capsicum annuum* L.), there were 758 DEGs, of which 573 were up-regulated and 185 were down-regulated (Fig. 2b). Annotation information for the DEGs in pepper is displayed in Additional File 4: **Table S4**. The most significantly up-regulated genes in pepper were involved in laccase, transcription regulator, protease inhibitors, proline dehydrogenase, reticulase, glutathione transferase, chaperones, and, etc. The most significantly down-regulated genes were involved in expansin, WAT1 related protein, bidirectional sugar transporter, vacuolar iron transporter homologue, and carbohydrate esterase, etc.

### Verification of selected DEGs using RT-qPCR

To verify the accuracy of the RNA-seq data, selected genes in *P. polymyxa* SC2 and pepper were subjected to RT-qPCR amplification.

**Fig. 3:**
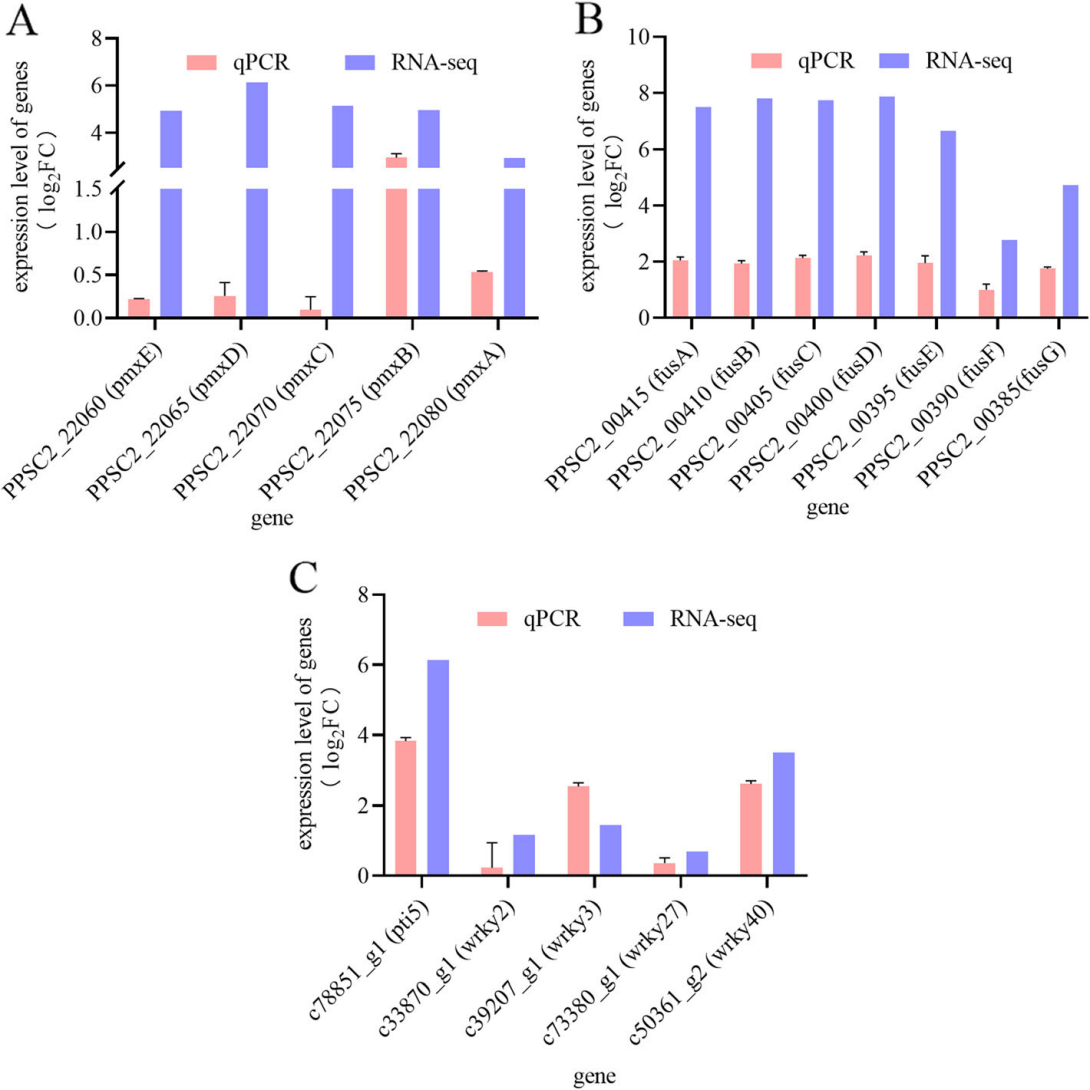
RT-qPCR of genes in *P. polymyxa* SC2 and pepper. RT-qPCR of polymyxin synthesis genes (**a**) and fusaricidin synthesis genes (**b**) in *P. polymyxa* SC2, and selected transcription factor genes in pepper (**c**). *GAPDH* housekeeping genes were used as reference genes. Relative expression levels were calculated using the ΔΔCt method. Values in RT-qPCR indicate means ± SD (*n* = 3), and values in RNA-seq indicate means (*n* = 3)

In RNA-seq, the genes (*pmxA*, *pmxB*, *pmxC*, *pmxD*, and *pmxE*) related to polymyxin synthesis in *P. polymyxa* SC2 were up-regulated by 2.93-, 4.95-, 5.13-, 6.13-, and 4.93-fold, respectively. In the RT-qPCR, compared with the control group, the expression of *pmx*A/B/C/D/E genes were also up-regulated in the treated group (Fig. 3a). A gene cluster involved in fusaricidin biosynthesis was also detected in *P. polymyxa* SC2. In the RT-qPCR, relative expression levels of genes in this cluster (*fusA*, *fusB*, *fusC*, *fusD*, *fusE*, *fusF*, and *fusG*) were significantly higher in the treatment group than in the control group (Fig. 3b), and in RNA-seq of *P. polymyxa* SC2, these genes were up-regulated 7.5-, 7.81-, 7.74-, 7.88-, 6.65-, 2.77-, and 4.73-fold, respectively. In the RNA-seq of pepper, some genes encoding transcription factors and genes related to disease resistance were changed in varying degrees. RT-qPCR results of pepper treated with *P. polymyxa* SC2 revealed that expression of the genes *wrky2*, *wrky3*, *wrky27*, *wrky40*, and *pti5* was up-regulated (Fig. 3c), congruent with the RNA-seq results. Overall, the expression trend of the selected genes in RT-qPCR was consistent with that in RNA-seq, indicating that the RNA-seq data were reliable.

### Overall analysis of DEGs in *P. polymyxa* SC2

**Fig. 4:**
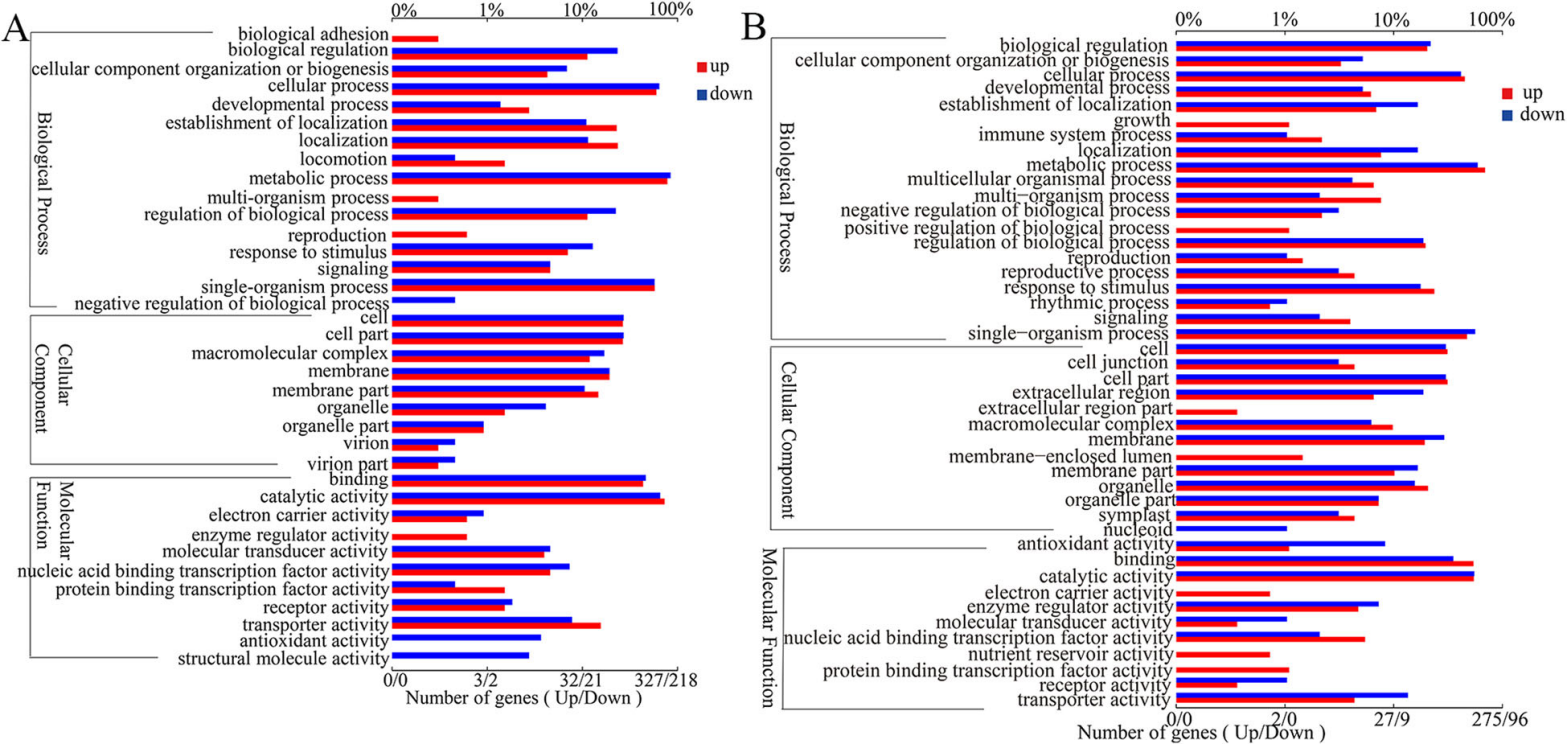
Enriched Gene Ontology (GO) terms distributed to the DEGs in *P. polymyxa* SC2 (**a**) and pepper (**b**). DEGs from *P. polymyxa* SC2 interacting with pepper, as identified by RNA-Seq, were enriched based on the GO database. The abscissa below the figure indicates the number of genes annotated to a GO term. The upper abscissa indicates the proportion of the number of genes annotated to a GO term to the total number of all GO-annotated genes. (Genes and GO terms are many-to-many relationships; a gene can contain multiple GO term annotations, and a GO term can also correspond to multiple genes, not one-to-one relationships). Ordinates represent each detailed classification of GO. Three squares represent three secondary classifications of GO, respectively

DEGs of *P. polymyxa* SC2 were mainly distributed in 36 sub-classes of three major categories in the GO database (Fig. 4a). Many genes were classified into cellular process, metabolic process, single-organism process, binding, and catalytic activity classes. Up-regulated genes were predominantly in the classes of enzyme regulator activity, biological adhesion, and multi-organism process, while down-regulated genes were associated with negative regulation of the biological process, antioxidant activity, and structural molecule activity. About 65 genes were significantly enriched into molecular function term. And there were 12, 12, 16, 13, and 12 genes enriched into phosphopantetheine binding term, modified amino acid binding term, amino acid binding term, vitamin binding term, and amide binding term, respectively. There were 61 genes enriched into biological process term. About 21, 18, 11, and 11 genes were significantly enriched into tetrapyrrole metabolic process, tetrapyrrole biosynthetic process, cobalamin biosynthetic process, cobalamin metabolic process, respectively.

**Fig. 5:**
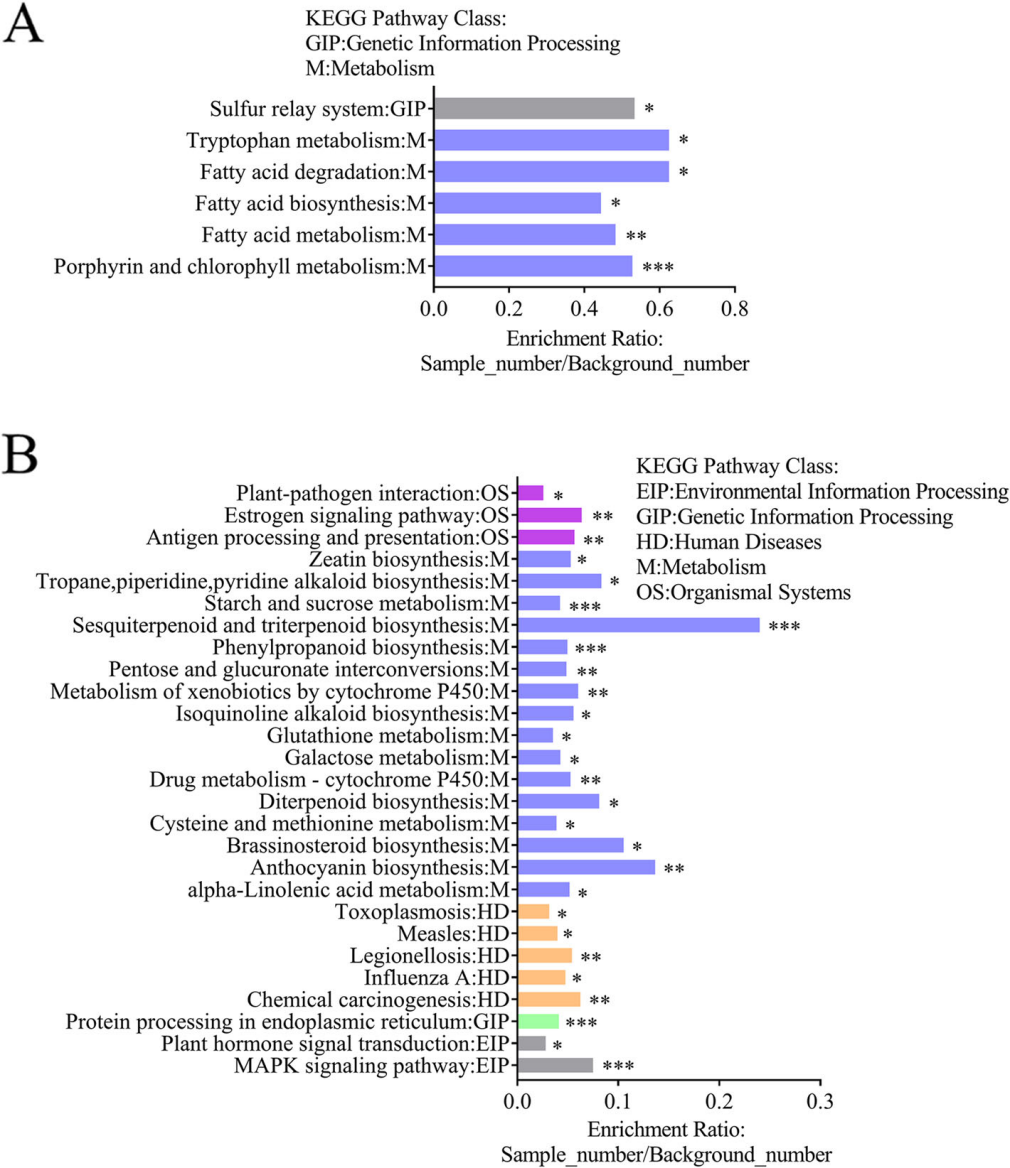
KEGG Enrichment of DEGs in *P. polymyxa* SC2 (**a**) and pepper (**b**). DEGs were also enriched according to the KEGG database [29]. Each column presents a path, and ordinate text indicates the name and classification of the path. The height of a column is expressed in ordinates enrichment rate (Enrichment Ratio = Sample Number/Background Number). *** *p* < 0.001, ** *p* < 0.01, and * *p* < 0.05

DEGs were also enriched according to the KEGG database. Results for *P. polymyxa* SC2 are shown in Fig. 5a. Numerous genes were enriched in various categories connected to metabolism, including 60 genes in the metabolism of amino acids and other amino acid classes of the metabolism category; 91 genes in carbon metabolism; 28 in energy metabolism; 25 associated with lipid metabolism; and 43 genes in the metabolism of cofactors and vitamins. A total of 63 genes were significantly enriched into the metabolism pathway and genetic information processing pathway. There were 19, 14, 12, 5, 5 genes enriched into porphyrin and chlorophyll metabolism pathway, fatty acid metabolism pathway, fatty acid biosynthesis pathway, fatty acid degradation pathway, and tryptophan metabolism pathway, respectively. Meanwhile, eight genes were enriched into sulfur relay system pathway which belongs to genetic information processing. Many genes were not enriched significantly. Transport genes were also up-regulated in *P. polymyxa* SC2. These included genes related to sulfate (*cysW*, *cysT5*, *cysT3*), molybdate (*modA1*, *modA3*), glycine (*proV*), and betaine (*PPSC2_06215*) transport in the mineral and organic ion transport classes. Up-regulation of genes associated with the transport of inorganic salt ions and minerals is beneficial for the absorption of inorganic salt ions and minerals in *P. polymyxa* SC2. A total of 43 genes related to ABC transport were detected in *P. polymyxa* SC2. ABC transport system genes associated with phosphate and amino acid transport (*glnP1, glnP3, occM3, hisP, phnE, ptxC, and pstB5*) were significantly up-regulated. Genes related to glutamine-transport (*glnP1*, *glnP3*) and cystine-transport (*occM3*, *hisP*) were up-regulated, as well as genes related to iron complexes, zinc/manganese/iron, and biotin transport in the metal cations, siderophores, and vitamin B12 transport category. Iron complex transport-related genes (*fhuC1*, *fhuD1*, *fhuG7*, *fhuC3*, *yclP*, *yxeB11*, *yfmD*, and *cbrA1*) were also up-regulated in varying degrees. Up-regulation of all these genes enhances the ability of *P. polymyxa* SC2 to transport metal ions. Metal ions have significant roles in the function of enzymes, which will be involved in many biological processes. Thus, the ability of *P. polymyxa* SC2 to transport ions will be beneficial to its growth.

### Overall analysis of DEGs in peppers

Inoculation of pepper with *P. polymyxa* SC2 led to some changes in gene expression in pepper. DEGs in pepper were mainly distributed in 44 sub-categories of three main categories in the GO database (Fig. 4b). There were abundant genes distributed in the classes of metabolic process, catalytic activity, binding, single-organism process, and cellular process. About 1110, 35, and 1069 genes were significantly enriched into biological process, cellular component, molecular function terms, respectively. A total of 307, 177, and 141 genes were enriched into biological process, single organism process, and single organism metabolic process, respectively. Thirty-five genes were significantly enriched into extracellular region which belongs to the cellular component. For molecular function, there were 315 and 203 genes enriched into molecular function and catalytic activity terms, respectively. The expression of many genes related to membrane-enclosed lumen, growth, positive regulation of the biological process, protein binding transcription factor activity, electronic carrier activity, nutrient reservoir activity, and extracellular region part related functions were up-regulated.

A total of 276 DEGs of pepper were enriched by KEGG analysis (Fig. 5b). A total of 105 genes were significantly enriched into five pathways, such as organismal systems (12 genes), metabolism (55 genes), human diseases (19 genes), genetic information processing (13 genes), environmental information processing (6 genes). These included 15 genes involved in the mitogen-activated protein kinase (MAPK) signaling pathway (6 genes) and plant hormone signal transduction (9 genes); 153 genes involved in metabolism; 12 genes involved in sesquiterpenoid and triterpenoid biosynthesis; and 11 genes related to phenylpropanoid biosynthesis.

### Correlation between functional genes of *P. polymyxa* SC2 and pepper

#### Mutual recognition, chemotaxis, and colonization ability of *P. polymyxa* SC2 with pepper

Under the stimulation of pepper, a total of 19 genes related to the quorum sensing in *P. polymyxa* SC2 were up-regulated (Table 2). Up-regulated expression of genes related to quorum sensing could help *P. polymyxa* SC2 perceive environment changes. Expression of the gene *PPSC2_08335*, encoding chemotactic protein AER, was up-regulated by 3.25-fold, and this is likely to benefit *P. polymyxa* SC2 in receiving external signals and responding to environmental changes. Correlation analysis revealed that genes involved in histidine metabolism (*c66011_g2*), glutamic acid metabolism (*c39553_g2*), phenylalanine/tyrosine/tryptophan biosynthesis (*c119522_g1*), amino sugar/nucleotide glycogen metabolism (*c48054_g1*), alpha-linolenic acid metabolism gene (*c79159_g1*) in pepper associated with the gene encoding AER protein in *P. polymyxa* SC2. Pepper not only stimulated the expression of chemoreceptors but also affected the expression of specific chemotaxis genes in *P. polymyxa* SC2. These included genes such as *cheA, cheW, cheY, cheD*, and *cheC*, which were up-regulated by 1.49- to 2.11-fold. Meanwhile, two genes (*fliM* and *fliN*) encoding flagellar motor switch proteins were both up-regulated by 2.16-fold. This indicated that in the presence of pepper, the motility of *P. polymyxa* SC2 was enhanced. This would be conducive to the colonization of *P. polymyxa* SC2 in the pepper rhizosphere.

**Table 2:** DEGs related to quorum sensing, chemotaxis, and biofilm formation in *P. polymyxa* SC2

	Gene	Product	name	log_2_FC(SH/S)
Quorum sensing	*PPSC2_02180*	peptide ABC transporter substrate-binding protein	*oppF1*	2.61
	*PPSC2_02185*	peptide ABC transporter permease	*oppB1*	4.2
	*PPSC2_02190*	peptide ABC transporter permease	*oppC1*	3.94
	*PPSC2_02195*	ABC transporter substrate-binding protein	*oppA1*	4.92
	*PPSC2_05715*	peptide ABC transporter substrate-binding protein		2.65
	*PPSC2_05720*	transports peptides consisting of two or three amino acids	*dppB*	2.71
	*PPSC2_05725*	peptide ABC transporter permease	*oppC3*	2.31
	*PPSC2_10965*	ABC transporter substrate-binding protein	*oppA5*	2.11
	*PPSC2_10970*	diguanylate cyclase	*oppB5*	2.2
	*PPSC2_10975*	peptide ABC transporter permease	*oppC5*	2.85
	*PPSC2_10980*	peptide ABC transporter ATPase	*oppD3*	2.88
	*PPSC2_10985*	peptide ABC transporter substrate-binding protein	*oppF3*	2.58
	*PPSC2_13025*	nickel transporter permease NikC	*dppC3*	2.23
	*PPSC2_24700*	ABC transporter ATPase	*oppD7*	2.07
	*PPSC2_25400*	peptide ABC transporter substrate-binding protein	*oppF9*	2.94
	*PPSC2_25405*	peptide ABC transporter ATPase	*oppD9*	3.6
	*PPSC2_25410*	peptide ABC transporter permease	*oppC9*	3.35
	*PPSC2_25415*	diguanylate cyclase	*oppB9*	3.26
	*PPSC2_25420*	ABC transporter substrate-binding protein	*dbp*	2.91
Chemotaxis	*PPSC2_08335*	chemotaxis protein AER	*aer*	3.25
	*PPSC2_18805*	chemotaxis protein		−4.65
	*PPSC2_09975*	histidine kinase CheA	*cheA*	2.11
	*PPSC2_09985*	cheC	*cheC*	1.71
	*PPSC2_09990*	chemotaxis protein CheD	*cheD*	1.49
	*PPSC2_14595*	chemotaxis protein CheR	*cheR3*	2.73
	*PPSC2_21235*	chemotaxis protein CheR	*cheR5*	−2.53
	*PPSC2_09980*	coupling protein CheW	*cheW*	2.04
	*PPSC2_09970*	effector protein CheY	*cheY*	2.26
	*PPSC2_09915*	flagellar motor switch protein FliM	*fliM*	2.16
	*PPSC2_09920*	flagellar motor switch protein FliN	*fliN*	2.16
Biofilm formation	*PPSC2_00125*	AbrB family transcriptional regulator	*abrB*	−3.06
	*PPSC2_23095*	Transcriptional regulatory protein DegU	*degU*	2.02
	*PPSC2_05800*	tyrosine protein kinase	*epsB*	6.69
	*PPSC2_05810*	multidrug MFS transporter	*epsE*	6.25
	*PPSC2_25275*	transcriptional regulator	*sinR*	−1.2
	*PPSC2_03845*	histidine kinase	*kinA*	2.34
	*PPSC2_03710*	Regulator Abh	*abh*	2.74

After interacting with peppers, genes related to biofilm formation of *P. polymyxa* SC2 were changed in varying degrees. Expression of *kinA*, *epsB*, and *epsE* was up-regulated by 2.34- to 6.69-fold, while expression of s*inR* and *abrB* was down-regulated by 1.21-fold and 3.06-fold, respectively. The genes (*degU* and *abh*) related to biofilm formation were up-regulated by 2.02- and 2.74-fold. They may be indicated to promote biofilm formation for *P. polymyxa* SC2. In summary, pepper stimulated biofilm formation of *P. polymyxa* SC2, which would be conducive for colonization in the pepper rhizosphere.

### Growth-promoting analysis of potential nutrient supply between *P. polymyxa* SC2 and pepper

After interacting with peppers, expression of gene encoding phytase (*PPSC2_05715*) in *P. polymyxa* SC2 was up-regulated by 2.65-fold. Phytase can hydrolyze phosphate residues from phytic acid. The up-regulated expression of phytase may increase the concentration of inorganic phosphorous in the medium and promote the growth of peppers. In the RNA-seq of *P. polymyxa* SC2 interacted with pepper, some genes related to carbon metabolism and amino acid metabolism were detected in *P. polymyxa* SC2. Most of them were up-regulated (data are shown in Table 3). It seems clear that peppers provide some nutrients for the growth of *P. polymyxa* SC2.

**Table 3:** Genes related to metabolism in *P. polymyxa* SC2

Type	gene	product	log_2_FC(SH/S)
Fructose and mannose metabolism	*PPSC2_05870*	glycosyl hydrolase	2.69
	*PPSC2_05910*	GDP-mannose 4,6-dehydratase	7.84
	*PPSC2_05865*	mannose-1-phosphate guanylyltransferase	3.29
	*PPSC2_02440*	PTS friuctose transporter subunit IIB	3.46
	*PPSC2_24735*	rhamnose isomerase	−2.09
	*PPSC2_02450*	PTS mannose transporter subunit IID	2.2
	*PPSC2_05905*	GDP-L-fucose synthase	6.89
	*PPSC2_02445*	PTS mannose transporter subunit IIC	2.51
	*PPSC2_24740*	rhamnulose-1-phosphate aldolase	−2.43
	*PPSC2_02435*	PTS mannose transporter subunit IID	3.96
Starch and sucrose metabolism	*PPSC2_05860*	UDP-glucose 6-dehydrogenase	4.22
	*PPSC2_05940*	UDP-glucose 6-dehydrogenase	6.53
	*PPSC2_16055*	pectin methylesterase	3.4
	*PPSC2_03770*	levansucrase	3.25
	*PPSC2_05805*	UTP-glucose-1-phosphate uridylyltransferase	5.83
	*PPSC2_03430*	PTS beta-glucoside transporter subunit IIABC	−4.99
	*PPSC2_03425*	trehalose-6-phosphate hydrolase	−4.69
	*PPSC2_13470*	glucose-1-phosphate adenylyltransferase	−3.71
Glycerolipid metabolism	*PPSC2_09660*	phosphate acyltransferase	2.53
	*PPSC2_00395*	aldehyde dehydrogenase	6.65
	*PPSC2_15995*	glycerol dehydrogenase	−2.61
Alanine, aspartate and glutamate metabolism	*PPSC2_01185*	asparagine synthetase	4.66
	*PPSC2_25355*	adenylosuccinate synthetase	−2.86
	*PPSC2_14255*	aspartate ammonia-lyase	3.69
	*PPSC2_12155*	acetylornithine aminotransferase	6.28
	*PPSC2_06000*	asparagine synthase	5.58
	*PPSC2_22115*	glutamine--fructose-6-phosphate aminotransferase	−2.9
	*PPSC2_16495*	aspartate carbamoyltransferase	−2.11
Valine, leucine and isoleucine biosynthesis	*PPSC2_09040*	transferase	2.34
	*PPSC2_07085*	2-isopropylmalate synthase	2.76
	*PPSC2_07090*	3-isopropylmalate dehydrogenase	−2.59
	*PPSC2_00400*		7.88

### Defense mechanisms between *P. polymyxa* SC2 and pepper

Polymyxin and fusaricidin are important secondary metabolites of *P. polymyxa* SC2, and inhibit the growth of pathogenic bacteria and fungi, respectively. Under the stimulation of pepper, expression of genes related to polymyxin and fusaricidin biosynthesis in *P. polymyxa* SC2 was significantly up-regulated by 2.93- to 6.13-fold and 2.77- to 7.88-fold, respectively (Table 4). The *ectB* gene (*PPSC2_11845*) encoding aminotransferase for polymyxin production was up-regulated by 2-fold. Up-regulation of genes related to polymyxin biosynthesis may increase the production of polymyxin, which may strengthen the resistance of pepper to bacterial pathogens in nature. Genes related to fatty acid synthesis (*accB*, *fabG5*, *fabH3*, *fabD3*, and *fabG13*) were all up-regulated (3.74-, 2.43-, 2.93-, 3.31-, and 2.93-fold, respectively). The changes in these genes may be beneficial for the synthesis of fusaricidin because this process requires fatty acid side chains.

**Table 4:** DEGs related to secondary metabolic clusters in *P. polymyxa* SC2

	Gene	product	Name	log_2_FC(SH/S)
polymyxin biosynthesis	*PPSC2_22060*	polymyxin synthetase E	*pmxE*	4.93
	*PPSC2_22065*	multidrug ABC transporter permease	*pmxD*	6.13
	*PPSC2_22070*	multidrug ABC transporter permease	*pmxC*	5.13
	*PPSC2_22075*	ATP-dependent asparagine adenylase	*pmxB*	4.95
	*PPSC2_22080*	synthetase 2 Gramicidin S synthetase II	*pmxA*	2.93
fusaricidin biosynthesis	*PPSC2_00380*	membrane protein	*ymcC*	4.92
	*PPSC2_00385*	enoyl-ACP reductase	*fusG*	4.73
	*PPSC2_00390*	peptide synthetase	*fusF*	2.77
	*PPSC2_00395*	aldehyde dehydrogenase	*fusE*	6.65
	*PPSC2_00400*	acetolactate synthase large subunit	*fusD*	7.88
	*PPSC2_00405*	3-oxoacyl-ACP synthase	*fusc*	7.74
	*PPSC2_00410*	3-hydroxymyristoyldehydratase	*fusB*	7.81
	*PPSC2_00415*	bacitracin synthetase	*fusA*	7.5

**Fig. 6:**
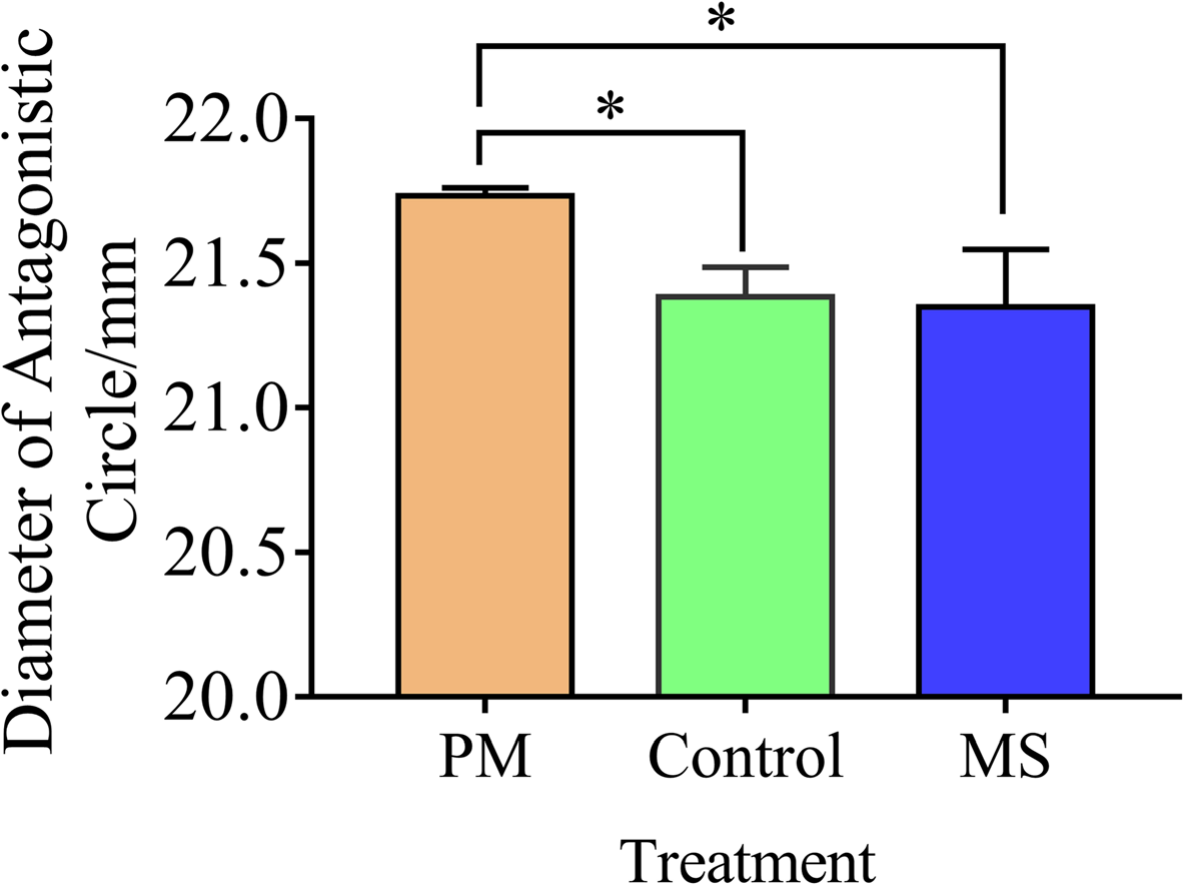
Antagonistic assay of *P. polymyxa* SC2 against *Xanthomonas citri*. The antagonistic assay comprised three treatment groups. Control: fermentation medium was inoculated *P. polymyxa* SC2. MS group: fermentation medium was supplemented with 1 mL MS medium and then inoculated with *P. polymyxa* SC2. PM group: fermentation medium was supplemented with 1 mL MS medium (which has been used for culturing peppers) and then inoculated with *P. polymyxa* SC2. Values indicate means ± SD (*n* = 3; * *P* < 0.05, Student’s *t*-test)

The antagonistic results of *P. polymyxa* SC2 against the pathogenic bacterium *Xanthomonas citri* were shown in Fig. 6. Fermentation broth of *P. polymyxa* SC2 supplemented with MS medium (which has been used for culturing pepper) had the best antagonistic effect on *X. citri.* The effects of *P. polymyxa* SC2 were also tested on the growth of *Fusarium moniliforme*, but the antagonistic circle in the treated group was smaller than that in the control group (data not shown).

While pepper influenced the expression of genes of *P. polymyxa* SC2, the bacterium also played an important role in the expression of pepper genes. *P. polymyxa* SC2 stimulated an up-regulation in expression of transcription factors (TFs) (Table 5), including *WRKY2* (*c33870_g1*), *WRKY3* (*c39207_g1*), *WRKY40* (*c50361_g2*), and *WRKY33* (*c66538_g3*), which were up-regulated by 1.17- to 4.2-fold, respectively. This finding suggests that *P. polymyxa* SC2 induces systemic resistance in pepper. Other pepper genes were also up-regulated after inoculation with *P. polymyxa* SC2. These included ethylene response factor 1 (ERF1) gene, up-regulated by 4.86-fold; the gene *c78851_g1* encoding Pti5, which can activate defense responses of plants to aphid and bacteria, up-regulated by 6.14-fold; genes directly involved in plant defense (such as *CML* and *PIK*1); genes involved in the jasmonic acid signaling pathway (*JAZ*), isoquinoline alkaloid biosynthesis (*TAT*), and phenylpropanoid biosynthesis (*MYB*); and genes *DNAJC7*, *PPID*, *HSPA1*, and *RNF5,* belonging to chaperone, heat shock protein, and ubiquitin, up-regulated by 4.12- to 5.86-fold. These results indicated that *P. polymyxa* SC2 could improve the defense ability of pepper.

**Table 5:** DEGs related to defense mechanisms in pepper

Gene	Name	log_2_FC (PH/P)	Gene	Name	log_2_FC (PH/P)
c119678_g1	*CML*	6.52	c118659_g1	*TAT*	4.68
c68413_g9	*CYP710A*	5.43	c33870_g1	*WRKY* 2	1.17
c48212_g2	*DNAJC7*	5.31	c73380_g1	*WRKY* 27	0.7
c56938_g1	*ERF1*	4.86	c39207_g1	*WRKY* 3	1.45
c55034_g2	*HSFF*	4.52	c50361_g2	*WRKY* 40	3.5
c48321_g3	*HSPA1*	4.29	c66538_g3	*WRKY33*	4.2
c69002_g1	*JAZ*	5.17	c119537_g1		4.67
c89163_g1	*MYBP*	5.28	c48656_g1		5.52
c68645_g1	*PIK1*	4.53	c68046_g1		6.74
c50209_g1	*PPID*	5.86	c98990_g1		−5.21
c78851_g1	*Pti5*	6.14	c99473_g1		4.02
c61710_g1	*RNF5*	4.12			

## Discussion

*P. polymyxa* SC2 promotes the growth of pepper and inhibits several phytopathogens [30]. To understand the interactional relationship of *P. polymyxa* SC2 and pepper, a pot experiment and transcriptome analysis of *P. polymyxa* SC2 and pepper in co-cultured conditions were conducted.

In the pot experiment, *P. polymyxa* SC2 not only increased the stem diameter and leaf size of pepper, but also significantly increased the chlorophyll content of pepper leaves. Chlorophyll participates in energy transfer by absorbing light energy in the process of photosynthesis [31]. PGPR can promote plant growth by increasing the chlorophyll content of plants. *Actinetobacter calcoaceticus* p23As, as a member of PGPR, significantly increased the chlorophyll content of monocot *Lemna minor* (duckweed) and the dicot *Lactuca sativa* (lettuce) [32]. The PGPR *Bacillus megaterium* M3 and *B. subtilis* OSU142, which were previously reported as plant-growth-promoting and potential biocontrol agents, could increase the chlorophyll content of wheat [33]. Chlorophyll contents of chickpea were significantly increased following inoculation with *P. polymyxa* [34]. Increasing the chlorophyll content may also increase the biomass of plants. However, in the current study, differences in biomass of pepper were not obvious in the group inoculated with *P. polymyxa* SC2 (data not shown). This may be because the growth of pepper in the pot is limited by space.

Interaction between *P. polymyxa* SC2 and pepper resulted in numerous differences in gene expression in the transcriptome of *P. polymyxa* SC2. Chemotaxis is advantageous for bacterial colonization in the rhizosphere of plants [35, 36], and can mediate beneficial bacteria-plant interactions [37]. After interacting with pepper, expression of chemotactic genes of *P. polymyxa* SC2 changed in varying degrees. Expression of genes encoding histidine kinase CheA and the coupling protein CheW was up-regulated by 2.11- and 2.04-fold, respectively. These genes help *P. polymyxa* SC2 respond to chemical stimuli. Up-regulation of effector protein CheY and the flagellar motor switch proteins FliM and FliN could promote motility of *P. polymyxa* SC2. These results indicated that pepper roots may secrete signaling molecules that can attract *P. polymyxa* SC2 to move towards pepper. Previous studies have reported that root exudate components can attract PGPR motility. Phazna et al. showed that six organic acids in the root exudates of *Capsicum chinense* were chemotactic for *Pseudomonas, Burkholderia*, and *Bacillus* [38]. Biofilm formation also plays an important role in the colonization of PGPR [39]. The proteins SinR and AbrB are negative regulators in biofilm formation [40]. After interacting with pepper, expression of the genes *sinR* and *abrB* was down-regulated in *P. polymyxa* SC2. This suggested that the root exudates may induce TasA operon expression [41]. Meanwhile, expression of genes related to biofilm formation (*kinA*, *epsB*, *epsE*, *degU*, and *abh*) in *P. polymyxa* SC2 was up-regulated by 2.02- to 6.69-fold, respectively. This may facilitate *P. polymyxa* SC2 colonization in the rhizosphere of peppers.

Phytase plays an important role in phosphate solubilization [42]. After interacting with pepper, genes encoding phytase were up-regulated in *P. polymyxa* SC2. This indicated that *P. polymyxa* SC2 may enhance the ability of pepper to absorb phosphorus. Improving phosphorus uptake may promote pepper growth to some extent. Previous studies also found that phytase-secreting bacteria can enhance the phosphorous content in plants [43]. Pepper also stimulated the expression of metabolic genes in *P. polymyxa* SC2. In the RNA-seq of *P. polymyxa* SC2, various genes related to metabolism were up-regulated, including genes involved in fructose and mannose metabolism, which were up-regulated by 2.20- to 7.84-fold. Vančura and Hovadík reported that sucrose and fructose were components of red pepper root exudates [44], and this would explain the results of our study. Pepper also stimulated the expression of genes related to alanine, aspartate, and glutamate metabolism. We found that alanine could be used as a nitrogen source for growth of *P. polymyxa* SC2 growth (unpublished data). Together, these results suggest that root exudates of pepper may provide nutrients for the growth of *P. polymyxa* SC2.

In the presence of pepper, the expression of genes related to polymyxin and fusaricidin in *P. polymyxa* SC2 was up-regulated. Expression of the aminotransferase gene *ectB* was also up-regulated. L-2,4-diaminobutyric acid (L-Dab), the precursor of polymyxin synthesis, is synthesized by EctB [45]. In the initial stage of polymyxin synthesis, increasing Dab can increase the yield of polymyxin E [46]. However, after fermentation for 35 h, addition of L-Dab inhibited the production of polymyxin and suppressed *ectB* expression [45]. In this study, *P. polymyxa* SC2 interacted with pepper for 20 h before transcriptome sequencing. This short culture time may account for the increase in endogenous precursor Dab increasing the yield of polymyxin. In the antagonistic test, the antagonistic ability of *P. polymyxa* SC2 was confirmed to be stronger after adding the medium that had been used to culture pepper. This may be related to the up-regulated expression of polymyxin biosynthesis gene cluster in *P. polymyxa* SC2 under the effects of pepper root exudates. Although the expression of genes related to fusaricidin biosynthesis cluster was up-regulated, the ability of *P. polymyxa* SC2 to inhibit fungi (*F. moniliforme*) did not change significantly after adding the medium that had been used to culture pepper. This may be because polymyxin and fusaricidin compete for the same transporter genes to be secreted [47]. It is also possible that the MS medium contains substances that inhibit the synthesis of fusaricidin. Thus, genes related to fusaricidin biosynthesis in *P. polymyxa* SC2 were only up-regulated at the level of gene transcription.

For plants, WRKY is a superfamily of transcription factors that play important roles in many biological processes [48–53]. In the current study, the genes encoding WRKY2 and WRKY40 (*c33870_g1* and *c103783_g1*, respectively) were up-regulated in pepper. For the pepper, *C. annuum*, the gene encoding WRKY2 (*CaWRKY2*) was regarded as an early component of defense signaling and it was rapidly induced following inoculation with host or non-host pathogens [54]. The gene *CaWRKY40* was reported to be regulated by salicylic acid, jasmonic acid, and ethylene signaling in response to *Ralstonia solanacearum* infection and heat stress in pepper [55]. Combining our data with the above research leads us to speculate that *P. polymyxa* SC2 could induce the systemic resistance of pepper and enhance the resistance of pepper to some pathogens.

## Conclusion

In this study, *P. polymyxa* SC2 effectively improved the agronomic characteristics of peppers. The root exudates of pepper enhanced the antagonistic ability of *P. polymyxa* SC2 against pathogenic bacteria. Meanwhile, based on the transcriptomics data, pepper can induce the expression of genes related to polymyxin biosynthesis. Pepper could stimulate the expression of genes related to quorum sensing, chemotaxis, and biofilm formation in *P. polymyxa* SC2. Concurrently, *P. polymyxa* SC2 may also induce the systemic resistance of pepper by stimulating the expression of some TFs. This interactional relationship between pepper and *P. polymyxa* SC2 is the result of multiple pathways and coordinated regulation of various reactions. This study described the growth-promoting effects of *P. polymyxa* SC2 on pepper and contributes to elucidating the growth-promoting mechanisms of *P. polymyxa*.

## Methods

### Strains and plants

*P. polymyxa* SC2 was isolated from the rhizosphere soil of pepper in Guizhou, China and stored at 4 °C in the dark. Strain SC2 was activated on Luria-Bertani (LB) agar plates and then cultured at 37 °C for 24–48 h. A single colony of the strain was inoculated in 5 mL LB liquid medium, shaken at 37 °C overnight, then 5 mL was subcultured into 50 mL fresh LB and shaken at 37 °C for a further 12 h. Cell suspensions, diluted to ∼10^8^ cells/mL, were used for pot experiments. Cells were collected by centrifugation and were resuspended in 1× PBS buffer to an OD_600_ of 1.0. It was used for co-cultivation with pepper in a sterile environment. *Xanthomonas citri*, a pathogen causing citrus canker [56, 57], was used in the antagonistic test. *Xanthomonas citri* was inoculated on LB agar and cultured at 30 °C for 24–48 h. A single colony of *Xanthomonas citri* was then inoculated into 5 mL LB liquid medium and shaken at 30 °C overnight for the test. Pepper seeds (*Capsicum annuum* L. (Shengfeng)) were purchased from Nongda Seed Company, Tai’an, China.

### Promotion effects of *P. polymyxa* SC2 on pepper in pot conditions

Pot experiments were conducted in the greenhouse of Shandong Agricultural University. Approximately four pepper seeds were planted in each hole of aperture disks containing vermiculites, and were cultured at 25 °C. After germination, a seedling remained in each hole. Seedlings with 4–5 euphylla were ready for pot experiments. Approximately 3 kg healthy soil (or continuous cropping soil) was placed in a pot (25 cm diameter and 15 cm depth). Pepper seedlings in a similar growth trend were selected and one seedling was transplanted into each pot and irrigated with 500 mL Hoagland nutrient solution [58]. Experimental treatments commenced after 3 days. Treated group: each pot of peppers was irrigated with 5 mL *P. polymyxa* SC2 fermentation broth (1 × 10^8^ CFU/mL) and diluted with water to 200 mL. Control group: 5 mL sterilized LB medium was poured into the rhizosphere soil of peppers in the same way as the treated group. Fifteen biological replicates were set per treatment. Agronomic traits including stem diameter (diameter at the ground base), plant height (vertical height from the soil surface to the highest point of the main stem), leaf width, and leaf length were investigated at 30, 40, and 50 dpi. Fresh weights of above ground and underground parts were determined after pot experiments. At 40 dpi, the leaves of five pepper plants were randomly selected for chlorophyll determination by the ethanol method [59].

### Interaction treatments of *P. polymyxa* SC2 and pepper in sterile conditions

To better analyze the interaction mechanisms between *P. polymyxa* SC2 and peppers, a co-culture experiment was carried out under sterile conditions. To achieve these conditions, the peppers needed to be sterile. The surfaces of pepper seeds were sterilized by dipping in 75% (vol/vol) ethanol for 5 min, then the seeds were immersed in 1‰ (vol/vol) mercury dichloride for 20 min before rinsing with sterile distilled water for 5–7 times. Sterilized seeds were placed on wire mesh in tissue culture vessels containing Murashige and Skoog (MS) liquid medium prepared according to Guan’s method [60]. Culture vessels were then placed in a plant growth chamber at 25 °C with 16 h light (day) period (13,200 lx) and 8 h dark (night) period. Twenty days post-germination, seedlings were used for the interaction experiment, and at this time, roots of the seedlings grow in the liquid MS medium. *P. polymyxa* SC2 was inoculated into liquid medium to study the possible interactions between pepper and *P. polymyxa* SC2. The bacteria negative control comprised 1 mL *P. polymyxa* SC2 suspension applied to sterilized MS medium; *P. polymyxa* SC2 cells were collected after 20 h and named as [S]. The plant negative control comprised pepper seedlings treated with 1 mL of 1× PBS buffer; roots of the pepper seedlings were collected after 20 h and marked as [P]. The *P. polymyxa* SC2-pepper co-cultured group comprised peppers treated with 1 mL *P. polymyxa* SC2; bacterial cells and pepper roots were collected after 20 h and named as [SH] and [PH], respectively. Each treatment was designed with three biological replicates. The collected bacteria and pepper roots were frozen in liquid nitrogen and stored at − 80 °C until further processing.

### RNA extraction and RNA-seq

Total RNA of *P. polymyxa* SC2 and peppers was extracted and purified using the TRIzol (Invitrogen) method. Total RNA quantity and quality were assessed using a NanoDrop 2000 spectrophotometer. RNA integrity number (RIN) was investigated using an Agilent 2100 Bioanalyzer. RNA quality is an essential factor in RNA-seq, therefore only RNA samples with RIN > 6, 230/260 and 260/280 ratios> 2 were used. The mRNA of pepper was isolated from the crude RNA via Oligo (dT) according to the manufacturer’s (NEBNext® Ultra™ RNA Library Prep Kit for Illumina®) instructions. The mRNA of *P. polymyxa* SC2 was isolated by removing rRNA. The mRNA was randomly fractured into small fragments of approximately 200 bp, and was reversed to a single strand of cDNA by random primers. Two-strand cDNA was further synthesized to form a stable double-strand structure. End Repair Mix was used to complement double-stranded cDNA into flat ends, and then an A base was added at the 3′ end. The library was enriched and sequencing of cDNA fragments was conducted using an Illumina Hiseq4000 platform.

### Bioinformatics analyses

Raw RNA-seq data were stored in a fastq file format. To ensure the accuracy of subsequent bioinformatics analysis, raw reads were filtered by SeqPrep (https://github.com/jstjohn/SeqPrep) and Sickle (https://github.com/najoshi/sickle). In this stage, the reads without inserting fragments due to the self-connection of connectors, and the reads with N ratio over 10%, and the reads less than 20 bp were removed. Then the high-quality clean data were assembled and aligned. Clean reads of *P. polymyxa* SC2 were mapped to the reference genome of *P. polymyxa* SC2 (https://www.ncbi.nlm.nih.gov/nuccore/NC_014622.2). Bowtie2 was used to align the clean reads and reference genome [61]. Since there was no reference genome for pepper, Trinity software (http://trinityrnaseq.sourceforge.net/, vision:trinityrnaseq-r2013-02-25) was used to assemble the short fragment sequences of pepper after obtaining high-quality sequencing data from RNA-seq [62], and predict the ORFs. The ORFs were searched through HMMER3, and the annotated proteins were aligned with NR, String, SwissProt, and KEGG databases to obtain corresponding annotation information through Blastx (Version 2.2.25, E value<1e^− 5^). The software edgeR was used to analyze differentially expressed genes [63]. GO enrichment analysis of differentially expressed genes was performed using GOATOOLS software (https://github.com/tanghaibao/GOatools) [64]. KEGG pathway enrichment was conducted with KOBAS [65]. Fisher’s exact test was used to analysis of GO/KEGG enrichment. The *p*-value (p_fdr) ≤ 0.05 indicated that the GO/KEGG function was enriched significantly.

### Expression profiling by RT-qPCR

One microgram of purified total RNA was used as a template for first-strand cDNA synthesis using an *Evo M-MLV* RT Kit with gDNA Clean for qPCR (Accurate Biotechnology (Hunan) Co., Ltd). Selected genes for each treatment were amplified to validate the RNA-seq results. These genes were selected from the DEG lists obtained for each condition. Primer sequences were designed using Beacon Designer 7 and are listed in Additional File 7: **Table S5**. The gene encoding glyceraldehyde-3-phosphate dehydrogenase (*GAPDH*) of pepper was used as the reference gene and *GAPDH* of *P. polymyxa* SC2 was used as an endogenous control. Relative expression levels were calculated using the ΔΔCt method [66]. Three biological replicates were used for real-time quantitative PCR.

### Antibacterial activity assay

An experiment was designed to test the effects of pepper root exudates on antibiotic production by *P. polymyxa* SC2. There were three treatment groups. Control: fermentation medium (sucrose 43.6 g/L, (NH_4_)_2_SO_4_ 6.66 g/L, CaCO_3_ 6.26 g/L, KH_2_PO_4_ 0.2 g/L, NaCl 0.2 g/L, MgSO_4_ 0.2 g/L) inoculated with *P. polymyxa* SC2. Treated group 1 (MS): fermentation medium supplemented with 1 mL MS medium and then inoculated with *P. polymyxa* SC2. Treated group 2 (PM): fermentation medium supplemented with 1 mL MS medium (which has been used for culturing peppers) and then inoculated with *P. polymyxa* SC2.

*P. polymyxa* SC2 was inoculated into 5 mL LB liquid medium and cultured at 37 °C, 180 rpm for 8–12 h, before being subcultured into the appropriate fermentation medium at 2% inoculation volume and incubated at 37 °C, 180 rpm for 72 h. For the antagonistic test, the *P. polymyxa* SC2 cultures were centrifuged at 12000 rpm for 10 min to remove cells, and the supernatants were used. Sterile water with 2% agar was added into Petri dishes and allowed to coagulate, then Oxford cups were placed in the Petri dishes. Next, 2 mL *Xanthomonas citri* cells were mixed with 200 mL LB medium containing 1% agar and cooled below 55 °C to prepare the plates. A total of 100 μL *P. polymyxa* SC2 culture supernatant was loaded into the well of the Oxford cup and incubated at 37 °C for 24 h to observe the growth inhibition effect on *X. citri*. All treatments had three replicates.

### Statistical analysis

Statistical analyses were performed using the Student’s *t*-test in SPSS 19.0. Columns were drawn using GraphPad Prism 7. *P* < 0.05 (*) in columns means there was a significant difference, and *P* < 0.01 (**) means there was an extremely significant difference.

## Supplementary Information

**Additional file 1.** Detailed statistics of reads mapping: Table S1 Mapping proportion statistics in RNA-seq of strain SC2, Table S2 Mapping proportion statistics in RNA-seq of peppers.

**Additional file 2: Fig. S1** PCA analysis of samples based on the gene expression level (FPKM) in RNA-seq.

**Additional file 3: Table S3** Annotation of differentially expressed genes in *P. polymyxa* SC2.

**Additional file 4: Table S4** Annotation of differentially expressed genes in pepper.

**Additional file 5: Fig. S2** Heatmap of DEGs in *P. polymyxa* SC2.

**Additional file 6: Fig. S3** Heatmap of DEGs in pepper.

**Additional file 7: Table S5** Primers for RT-qPCR.

## Data Availability

The raw data of the transcriptome has been uploaded to the Sequence Read Archive (SRA) database in National Center for Biotechnology Information (NCBI). The accession numbers are SRP242237 (https://trace.ncbi.nlm.nih.gov/Traces/sra/?study=SRP242237) and SRP242239 (https://trace.ncbi.nlm.nih.gov/Traces/sra/?study=SRP242239).

## Abbreviations

PGPR: Plant growth-promoting rhizobacteria
ACC: 1-aminocyclopropane-1-carboxylate
IAA: Indole-3-acetic acid
IAN: Indole-3-acetonitrile
dpi: Days post-inoculation
DEGs: Differential expression genes
GO: Gene Ontology
KEGG: Kyoto Encyclopedia of Genes and Genomes
TFs: Transcription factors
LB: Luria-Bertani
MS: Murashige and Skoog
OD: Optical density
RIN: RNA Integrity Number
