# Supplementary material for: Interactional mechanisms of Paenibacillus polymyxa SC2 and pepper (Capsicum annuum L.) suggested by transcriptomics
Source: BMC Microbiol. 2021 Mar 4;21:70. doi: 10.1186/s12866-021-02132-2 (PMC7931354; doi:10.1186/s12866-021-02132-2)
Supplement: Supplementary file 1 — Additional file 1. Detailed statistics of reads mapping: Table S1 Mapping proportion statistics in RNA-seq of strain SC2, Table S2 Mapping proportion statistics in RNA-seq of peppers. [file 12866_2021_2132_MOESM1_ESM.docx]

Table S1 Mapping proportion statistics in RNA-seq of SC2 strain

| Sample | Mapping Read/high quality reads | Proportion (%) |
| --- | --- | --- |
| S-1 | 20596320/22698580 | 90.74% |
| S-2 | 19732703/21516234 | 91.71% |
| S-3 | 20461238/22717714 | 90.07% |
| SH-1 | 25878962/27992384 | 92.45% |
| SH-2 | 21992481/23728992 | 92.68% |
| SH-3 | 30489148/32551458 | 93.66% |

Table S2 Mapping proportion statistics in RNA-seq of peppers

| Sample | Mapping Read/high quality reads | Proportion (%) |
| --- | --- | --- |
| P-1 | 26926380/29887282 | 90.09% |
| P-2 | 41818316/48308894 | 86.56% |
| P-3 | 40646854/46542918 | 87.33% |
| PH-1 | 39572424/46240158 | 85.58% |
| PH-2 | 48950668/56167804 | 87.15% |
| PH-3 | 43331318/49968568 | 86.72% |
