# Supplementary material for: Interactional mechanisms of Paenibacillus polymyxa SC2 and pepper (Capsicum annuum L.) suggested by transcriptomics
Source: BMC Microbiol. 2021 Mar 4;21:70. doi: 10.1186/s12866-021-02132-2 (PMC7931354; doi:10.1186/s12866-021-02132-2)
Supplement: Supplementary file 2 — Additional file 2: Fig. S1 PCA analysis of samples based on the gene expression level (FPKM) in RNA-seq. [file 12866_2021_2132_MOESM2_ESM.docx]

We also carried out Principal Components Analysis (PCA) analysis based on the gene expression level (FPKM) of all samples in RNA-seq. We found that the samples in the treated group (SH_1, SH_2, and SH_3) were greater similar. And the samples in the control group (S_1, S_2) were similar. Although Sample S_3 was far away from S_1 and S_2. The value of PC1 was 71.9%, and this indicated that the differences between the control group and the treated group were caused by the grouping. The samples of peppers were divided into two parts. The samples from each group were not clustered together tightly. However, the value of PC1 was greater than PC2, indicating the main factor for the difference of samples during grouping.


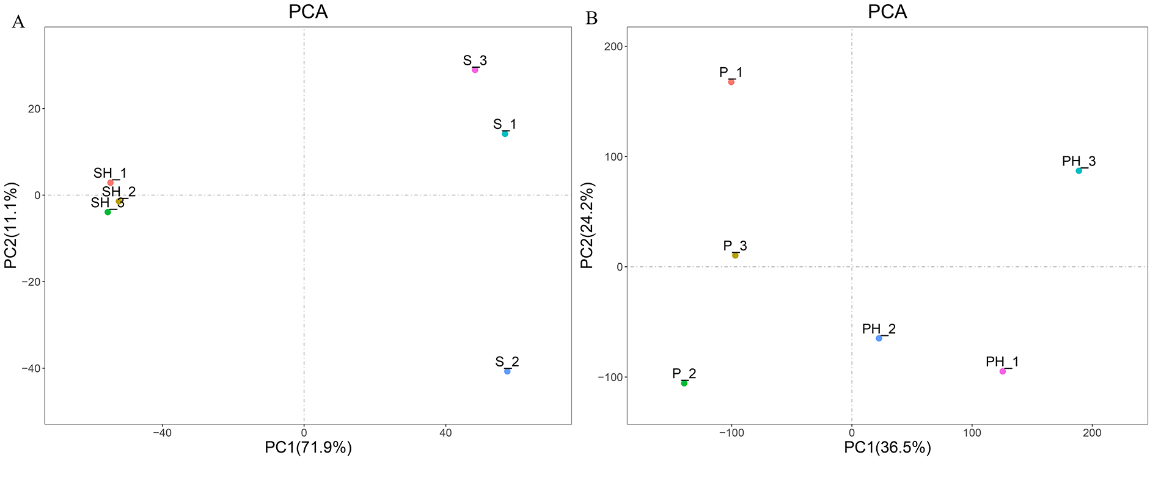


**Fig. S1 PCA analysis of samples based on the gene expression level (FPKM) in RNA-seq**

The PCA analysis based on the the gene expression level (FPKM) in RNA-seq was was performed using the OmicShare tools, a free online platform for data analysis (http://www.omicshare.com/tools). In this analysis, the data were normalized. A is the PCA analysis result of strain SC2 RNA-seq samples. B is the PCA analysis result of pepper RNA-seq samples.
