# Supplementary figures and images for: Interactional mechanisms of Paenibacillus polymyxa SC2 and pepper (Capsicum annuum L.) suggested by transcriptomics
Source: BMC Microbiol. 2021 Mar 4;21:70. doi: 10.1186/s12866-021-02132-2 (PMC7931354; doi:10.1186/s12866-021-02132-2)

Color Key

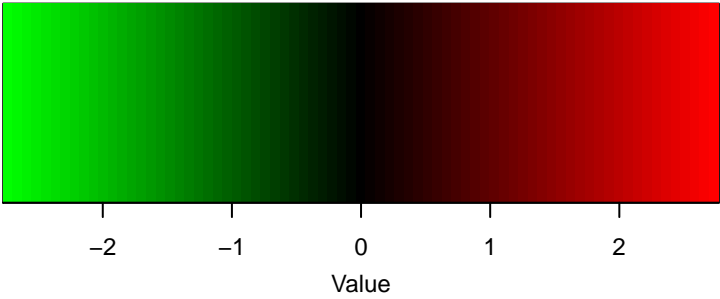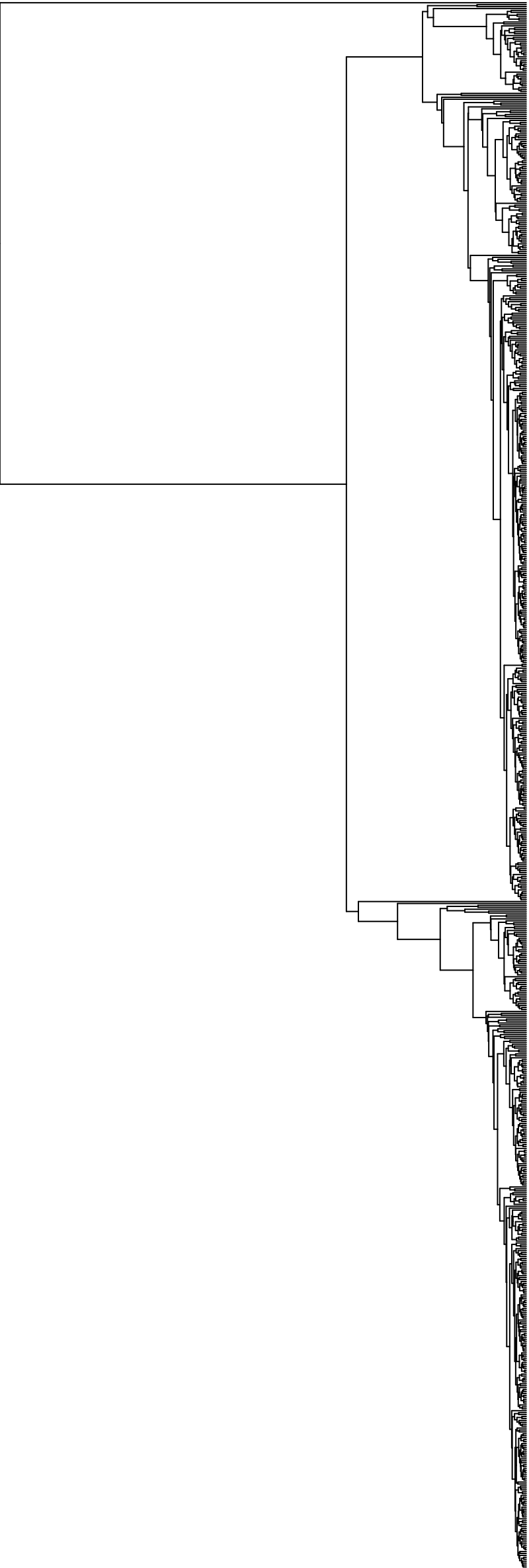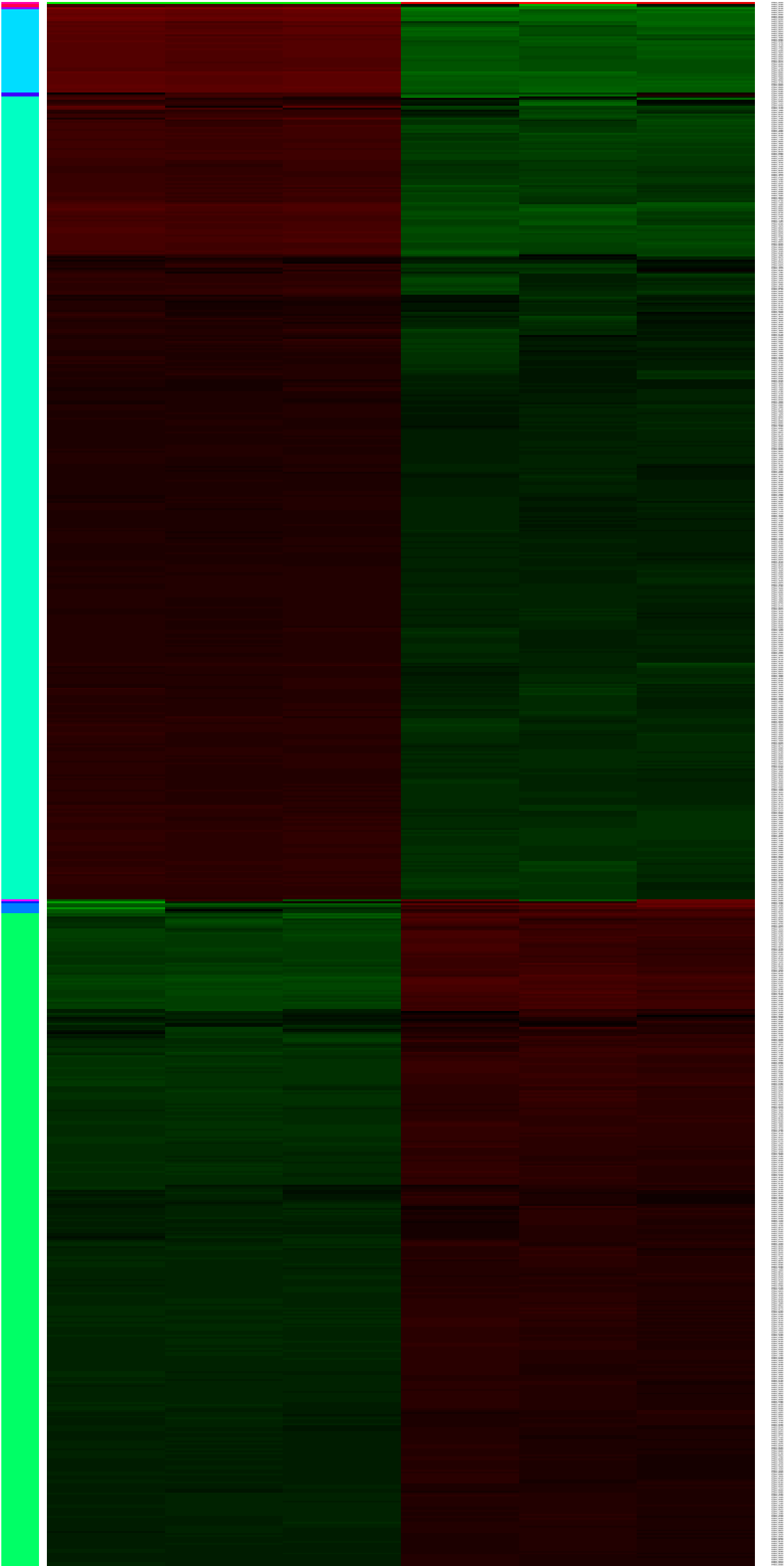

SH\_1

SH\_2

SH\_3

S\_2

S\_1

S\_3

Supplement: Supplementary file 5 — Additional file 5: Fig. S2 Heatmap of DEGs in P. polymyxa SC2. [file 12866_2021_2132_MOESM5_ESM.pdf]

Color Key

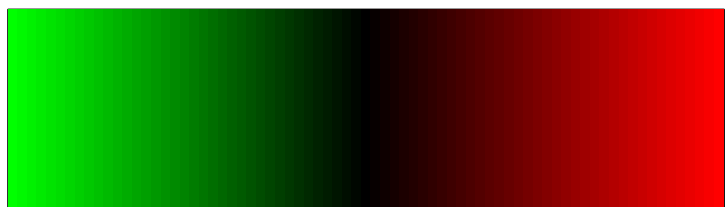

-1

Value

1

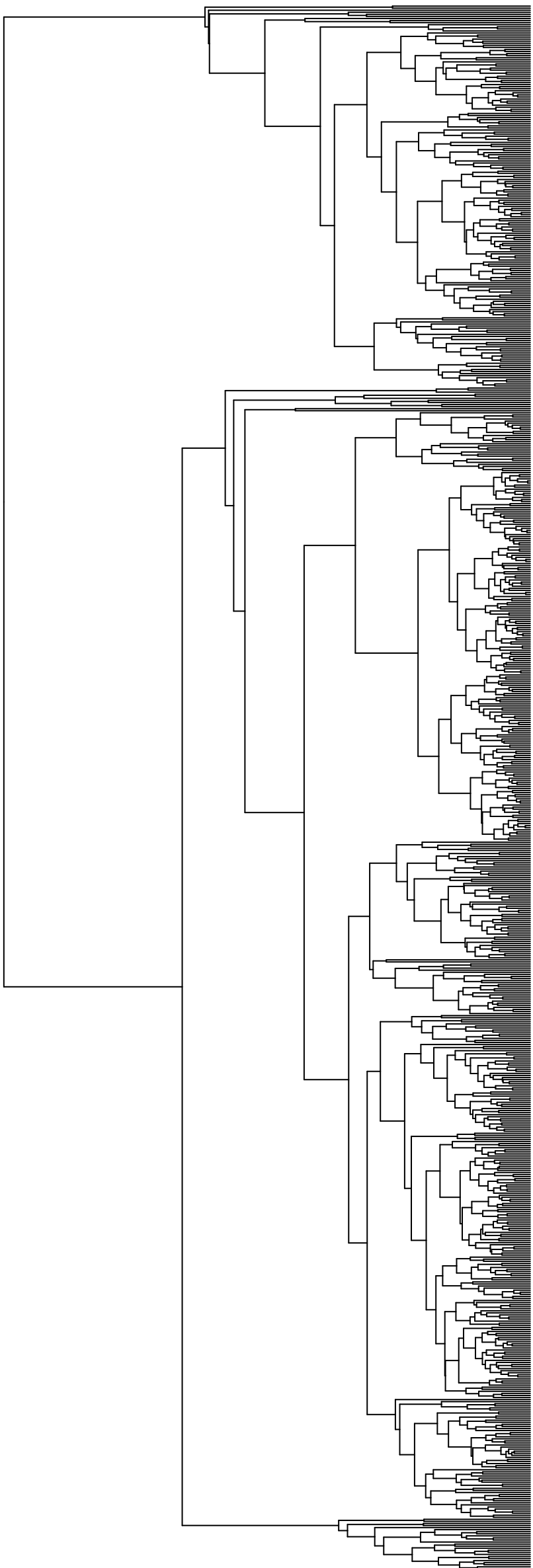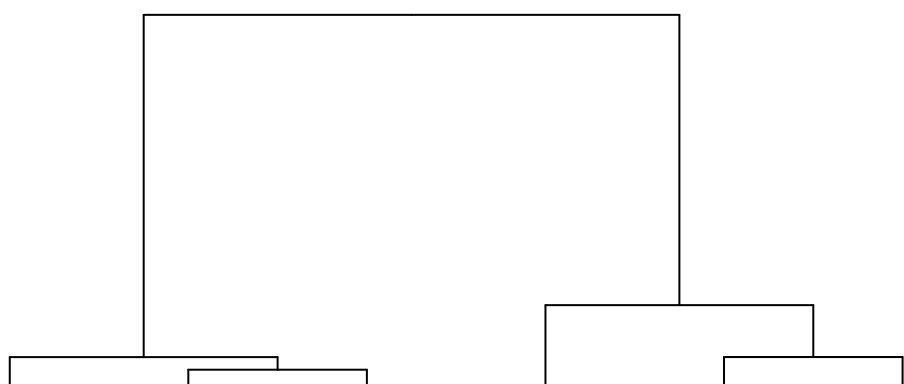

P\_3

P\_1

P\_2

PH\_3

PH\_1

PH\_2

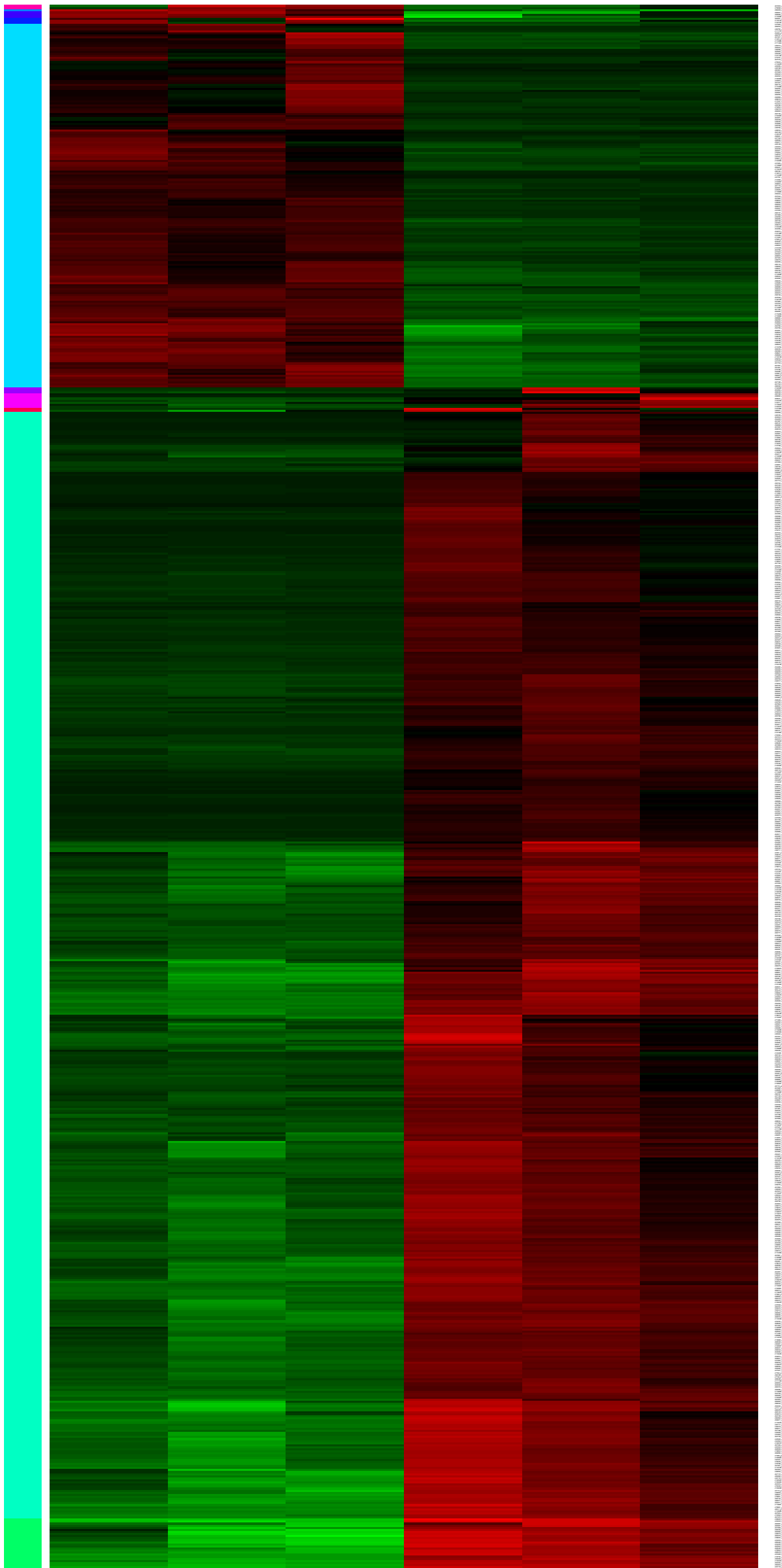

Supplement: Supplementary file 6 — Additional file 6: Fig. S3 Heatmap of DEGs in pepper. [file 12866_2021_2132_MOESM6_ESM.pdf]
